# Supplementary material for: Research on sublevel open stoping recovery processes of inclined medium-thick orebody on the basis of physical simulation experiments
Source: PLoS One. 2020 May 20;15(5):e0232640. doi: 10.1371/journal.pone.0232640 (PMC7239441; doi:10.1371/journal.pone.0232640)
Supplement: Attachment — Submitted filename: Suggestion_20200227.docx [file pone.0232640.s002.docx]

The experiment appears to have been carefully designed and executed, and the results should be of interest to the readership of PLOS ONE. However, before I can recommend publication, the author should address some issues, as follows:

1. The lack of all tables might not contribute to my review. Please upload tables. I strongly suggest the new manuscript should be reviewed again.

2. Please discuss how relevant the boundary condition is to existing conditions in the field. For example, in the mines considered, how the characteristics of overlying material (harder, softer, more or less dense) impact the results? Or would the mixture of unwanted material in an actual mine affect to a significant extent the experimental results?

3. There is not any information about the mechanical characteristics of the material. How the strength of the rock impact the results?

4. The paper is well written but still need English improvements. I advise the author to find a native English speaker to proofread the manuscript. The readability of the paper could be greatly improved, and this would make it much more accessible to the readership of PLOS ONE.
