# Supplementary material for: Research on sublevel open stoping recovery processes of inclined medium-thick orebody on the basis of physical simulation experiments
Source: PLoS One. 2020 May 20;15(5):e0232640. doi: 10.1371/journal.pone.0232640 (PMC7239441; doi:10.1371/journal.pone.0232640)
Supplement: Attachment — Submitted filename: Response to reviewers 4.16.doc [file pone.0232640.s003.doc]

**Authors’ reply to the Editor’s and Reviewers’ questions/comments/suggestions:**

The authors wish to greatly appreciate the invaluable and constructive comments, suggestions and contributions of the Editor and Reviewers to improve the quality of their research paper. All points highlighted by editor and reviewers have been fully addressed and clarified. Please find below the authors’ reply to the Editor’s and Reviewers’ comments/questions/suggestions:

**(Authors’ reply:** Blue in colour)

**Reviewer #1:**

1. Please include a copy of Tables 1-5 which you refer to in your text on page 6, 7 and 10.

**Authors:** Sorry for the mistake. Tables 1-5 have been added in the corresponding part of the text.

**Table1** Laboratory simulation of ore grain size composition

| In-situ Size/cm | <10 | 10~20 | 20~30 | 30~40 | 40~50 | 50~60 | 60~70 | 70~80 | 80~90 | 90~100 |
| --- | --- | --- | --- | --- | --- | --- | --- | --- | --- | --- |
| Test Size/mm | <2 | 2-4 | 4-6 | 6-8 | 8-10 | 10-12 | 12-14 | 14-16 | 16~18 | 18~20 |
| Ratio | 0.73 | 10.19 | 10.85 | 11.07 | 15.76 | 16.20 | 16.81 | 14.42 | 3.81 | 0.15 |

**Table2 Similarity factors of experiment model**

| Dimension(m) | Scaled ratio | Parameters of model |
| --- | --- | --- |
| Length(m) | 1:50 | 60 |
| Width(m) | 1:50 | 60 |
| Height (m) | 1:50 | 40 |
| Mining scheme (hours) | 1:7 | 2.5 |
| Bulk density (kg/m^3^) | 1:1 | 1.5×10^3^ |
| Ore-drawing shove (cm^3^) | 1:50 | 60 |

**Table 3** Experimental program design

| Index | Experimental program | |
| --- | --- | --- |
| 1 | Footwall side hole caving angle (°) | θ = 30/35/40/45/50/55 |
| 2 | Footwall friction coefficient (-) | f= 0.4/ 0.7/ 0.9 |
| 3 | Access drift interval (m) | d= 5/6/7.5 |
| 4 | Ore drawing burden (m) | w = 1/2/4 |

**Table 4** Dimension analysis parameters of ore simulation data set

| Number. | Parameter | Symbol | unity | Dimension |
| --- | --- | --- | --- | --- |
| 1 | Footwall blast angle | θ | ° | - |
| 2 | Footwall flatness | ƒ | - | - |
| 3 | Production drift spacing | d | m | L |
| 4 | Ore drawing burden | w | m | L |
| 5 | ore block length | L | m | L |

**Table 5** Dimension matrix predicted by recovery

| Parameter | Recovery | Footwall blast angle | Footwall Flatness | Production Drift Spacing | Ore Drawing burden | Ore Block Length |
| --- | --- | --- | --- | --- | --- | --- |
| F | 0 | 0 | 0 | 0 | 0 | 0 |
| L | 0 | 0 | 0 | 1 | 1 | 1 |
| 0 | 0 | 0 | 0 | 0 | 0 | 0 |

2. Please discuss how relevant the boundary condition is to existing conditions in the field. For example, in the mines considered, how the characteristics of overlying material (harder, softer, more or less dense) impact the results?

**Authors:** To guarantee that the results of a physical model can be directly scaled up, the model must be fully similar to the mine scale. Though physical model and full scale model can not meet the similarity in all aspects, geometric similarity is the most important and the easiest to implement in physical ore drawing simulation model.

(1) Conditions of similarity

To mimic the actual conditions in the field, the properties of materials used in the physical model were determined according to the following conditions of similarity:

a) Length scale C_L_

$$\begin{matrix} L_{\text{model}}=k\cdot L_{\text{mine}} \\ C_{\text{L}}=L_{\text{model}}/L_{\text{mine}} \\ C_{\text{L}}=k \end{matrix}$$

where k is the similarity coefficient. The value of k is equal to C_L_.

b) Time scale *C*_t_

By controlling the drawing speed, the similarity in granular particle movement process is determined. In the process of drawing, the movement distance of particles in the model is l in unit time. The particle motion process is the same, and the acceleration should be the same.

$$\begin{matrix} a_{\text{mine}}=a_{\text{model}} \\ L_{\text{mine}}:t^{2}_{\text{mine}}=L_{\text{model}}:t^{2}_{\text{model}} \end{matrix}$$

Accordingly,

The time of drawing operation satisfies the similarity coefficient.

C) Stress scale

According to the similar experimental conditions, the relationship between stress scale *C*_σ_ and length scale *C*_L_ and density scale *C*_ρ_ is as follows:

$$\begin{matrix} C_{\text{σ}}=C_{\text{ρ}}\cdot C_{\text{L}} \\ C_{\text{σ}}=C_{\text{L}}=k \end{matrix}$$

Through lab and in-site tests, the natural rest angle of the granules flow is consistent with the natural resting angle of the blasting ore, indicating that the mechanical conditions of the granules flow are consistent.

DLSOS mining method which is different from caving mining method (sublevel caving or block caving), belongs to open stope mining method. Its mining sequence is from bottom to top. Because the blast ore is mined under the protection of the top pillar, the blast muck is not affected by the volume weight of overlying strata, and the stress will not be transferred. Therefore, it is not necessary to apply the corresponding load on the upper part of the physical model.

(2) Determination of the parameters employed in physical model

The testing ore material for the physical simulation experiment is collected from the field. Therefore, the density （*C*_ρ_=1）and hardness of the ore material of the model are the same as those in the field. During the experiments, the test ore material is collected with the following procedure.

- the ore granularity is examined;
- the ore taken from the field will be crushed and screened according to the granularity listed in Table 1. And the ore will be collected by different particle sizes.
- given the set geometric similarity coefficient *k*, the gross mine weight *G* for simulation will be calculated. With the given abundant factor as 1.2, the ore volume with different granularity will be determined. The required weight of each particle size can be calculated according to the weight proportion of each particle size shown in Table 1. After weighing and mixing, the ore and waste rock meeting the requirements of particle size combination are prepared.

In the experiment, the artificial removal of ore was used to simulate the field ore transportation. The speed of one-time manual transportation is calculated according to the following process. According to the time similarity coefficient, the time to complete one ore drawing is determined.

The time of drawing operation satisfies the similarity coefficient: *k*^1/2^.

In mining experiment, the ore quantity of one blasting is 750 t, the efficiency of 3 m^3^ LHD is 500 t/shift, and the time of ore extraction after blasting is 1.5 shift. The mining time after blasting is 1.5 shifts. If each shift lasts 8 hours, the ore drawing time is 12 hours at a time.

The ore yield per hour is 62.5 t. According to the time similarity ratio, the length similarity ratio is *k* = 50, and *C*_t_ = 50^1/2^ = 7.07. Then the time of the physical ore drawing experiment when one burden is moved is calculated as below:

the time is 12/ *C*_t_ =12/7.07 = 1.697 h.

In the ore flow experiment, the loading capacity of the experimental model is 36.05 kg, with a total of 14 burdens. One burden loading capacity is 2.58 kg, and the drawing time is 1.697 hours. The ore drawing speed is required to be 25.34 g/min.

Therefore, the length, width and depth of the small shovel for drawing are 3 cm, 2 cm and 1 cm respectively. Ore draw quantity is 9g/circle and frequency is 2.8 times per minute. Based on the drawing frequency, the physical simulation drawing time can be guaranteed to be consistent with the in-site drawing time.

The physical simulation test ensures the consistency of time similarity and in-site experiment by controlling it as 2.58 times/min.

3. Or would the mixture of unwanted material in an actual mine affect to a significant extent the experimental results?

**Authors:** The mixing of waste rock has a great influence on the real industrial experiment results. In the actual mining production process, we do not want to mix waste rock into the ore, although the mixing of waste rock is inevitable. Because the mining method proposed in this paper is the open stope mining method, the prerequisite for the implementation of this mining method is that the stope is stable and the footwall and hang wall are stable. Therefore, the waste rock will not fall and mix into the ore falling in the room, thus reducing the quality of the ore. In the industrial experiment, the surrounding rock mass of the hanging and foot wall belongs to class I and II rock mass, which is of good quality. Under the selected structural parameters, the stope can remain stable without the rock falling from the hanging and foot wall and mixing into the ore.

In this paper, on the one hand, based on the ideal conditions of this mining method, waste rock does not mix in the ore. On the other hand, it is difficult to simulate the time and location of waste rock mixing in laboratory experiments. Therefore, in the selection of experimental indicators, the mixing rate of waste rock is not considered. And only the ore recovery rate is considered to study the location and quantity of mine loss, which provides support for the selection of parameters in industrial experiments.

This information is summarized on page 6, lines 187-202.

4. There is not any information about the mechanical characteristics of the material. How the strength of the rock impact the results?

**Authors:** This is a very good question. This problem involves a precondition of physical simulation experiment of bulk flow. The most important factor affecting ore flow in ore drawing experiment is particle size composition. The fluidity of ore particles with different sizes is different. The ore fluidity will directly affect the recovery quantity of ore, leading to the difference of the location and quantity of ore loss in the mining process. Thus it will cause the difference of mining efficiency and mining cost.

In the real mining, ore particles will collide with each other in the flow process. During the collision, the ore with low strength will be further broken, leading to the change of particle size distribution, and then affecting the flow behaviour of ore. However, this process usually occurs in the mining method or ore drawing pass with large ore rock movement distance. In the application of the mining method adopted in this paper, the movement of ore and rock is continuous and slowly downward. In the real production process, the possibility of large distance and high-speed collision of ore and rock is very small. In terms of the mine considered, the uniaxial compressive strength of the ore and rock is greater than 70MPa, and the uniaxial compressive strength of the rock is more than 40MPa. In this case, the rock is relatively strong, and there is little mutual expansion and fragmentation in actual production. Furthermore, the ore material used in the lab experiment is obtained directly from the site, thus the mechanical properties of the ore are the same as the real ore on the site. The particle grading is formulated according to the similarity ratio. The particles are relatively hard, and the collision will not lead to rock fragmentation.

Therefore, for the lab experiment, the impact of the mechanical properties of the ore on the recovery can be ignored.

5. The paper is well written but still need English improvements. I advise the author to find a native English speaker to proofread the manuscript. The readability of the paper could be greatly improved, and this would make it much more accessible to the readership of PLOS ONE.

**Authors:** As suggested, we have carefully checked the language of the manuscript including grammar and expression. With the help of a native English speaker in this major, the language has been polished.

Thank you for your constructive and valuable review comments.

**Reviewer #2:**

1. In chapter 2, the author should add some sketches to make "the sublevel open stoping using long-hole with back filling mining method" clearer.

**Authors:** Thank you for your suggestions. The original illustration of mining method (Figure 2) is a two-dimensional plan, which is not clear and intuitive enough when describing mining methods. As suggested, Figure 2 has been updated: the former planar graph remains as Figure 2 (b); and an additional three-dimensional map of mining method is added as Figure 2 (a). Fig. 2 (a) and Fig. 2 (b) are related by section lines I-I and Ⅱ-Ⅱ, which should be easier for readers to understand. This information is given on page 4 and 5, lines 106-144 and page 26 Fig.2(a)

(a)

（b）

**Fig. 2** The schematic of sublevel open stoping mining method with double-drilling roadway: (a) section sketch of mining method；(b) 3D model of the mining method.

1 Parallelogram stope; 2 Lower stope; 3 Upper stope; 4 Sublevel transport drift;

5 Drilling drift of lower stope; 6 Loading crosscut; 7 Sublevel filling drift; 8 Cutting raise of lower stope; 9 Drilling drift of upper stope; 10 Cutting raise of upper stope; 11 Fan drilling;

A: drift end losses; B: Ridge remnants; C: Sidewall remnants at footwall; D: Mixed waste rock at footwall

2. In chapter 3, the site conditions should be explained clearly in the similar simulation experiment.

**Authors:** Thank you for this good suggestion. The following information regarding the conditions of the site is provided on page 12 and 13, lines 393-396.

Jiaojia Gold Mine of Shandong Gold Group has a dip angle of 30°and a vertical thickness of 12m. The mineralization of the ore body is uniform and stable with regular geometry, which belongs to a typical inclined medium thick ore body. The quality of ore and rock can be classified as class I or II rock mass, and the stability of ore and rock is good. The sublevel open stopping using long-hole with back filling mining method is designed in this mine experiment. The mining parameters are determined according to the structural parameters from the above dimensional analysis: the length of the stope is L = 30m; the height of the section is 10m; the width of the stope, which equals to the thickness of the ore body, is 12M; the drift interval is d = 6m; the ore drawing ring burden is 2m; and the side hole angle of the footwall caving is 40 degrees. The parameters are consistent with the prototype parameters studied in the physical ore drawing simulation experiment, and the ore rock used in the experiment is directly taken from the mine experimental room; its particle size composition is also obtained based on the analysis of the real particle size of the mine. Through the above process, the similarity between the physical ore drawing simulation experiment and the real industrial experiment is ensured.

3. Please explain why the model is constructed with iron plate and acrylic plate and whether it will affect the simulation results.

**Authors:** (1) In the physical simulation experiment of bulk flow, the acrylic plate which is a white transparent material, is used as the external boundary of the model. During the experiment, the flow process of ore particles and the residual situation of ore can be observed from the outside of the model. Secondly, the acrylic plate characterized by high hardness, strength and toughness, is not easy to deform and suitable to process. Thus, the acrylic plate is employed in the construction of the ore drawing model.

The iron plate is used as a division plate of ring burden to simulate different blasting operations and to control the amount of ore caving. In the process of ore drawing experiment, the ring burden plate needs to be removed, so the rigidity and toughness of iron plate are suitable for drawing. Thus, iron plate is selected as the isolation plate in ore drawing experiment.

(2) In the industrial experiment, the rock surface of footwall formed by two adjacent blastholes is usually uneven. In the process of lab ore drawing physical experiment, in order to study the influence of the flatness of the footwall surface on the flow of the caving ore after blasting, the flatness of the footwall rock surface in actual production is simulated by placing the pasted fine sandpaper, coarse sandpaper and ore particles on the inner surface of the acrylic plate of the footwall (the side contacting the ore). The friction coefficient corresponding to different flatness is usually determined by the sliding experiment of inclined surface in classical mechanics. The method is shown in the figure below. Therefore, the acrylic material itself will not affect the ore fluidity.

In mining production, the blasting is carried out by upward medium deep holes. After blasting in medium deep holes, the blasting working faces (end position) of adjacent two rows are usually relatively flat. And when iron plate is used to simulate the division plate, the corresponding end position is the end position. Therefore, the influence of iron plate as the isolation plate on the ore fluidity can be ignored.

The friction coefficient of the acrylic plate with different pasted sandpapers and particles is calculated as follows:

The friction coefficients of fine sandpaper, coarse sandpaper, and mineral ore were determined by inclined sliding experiments. The experimental process is shown in the figure.

Test method for friction coefficient of different materials

The material to be tested is fixed on an inclined plane with an inclination of θ and can rotate around an axis. Standard cubes with 10 cm edge length and 1 kg surface smoothness are put on the inclined plane. We increase the angle of the inclined plane gradually and record the angle of the inclined plane when slider begins skating. The maximum static friction force F of the slider cube satisfies the formula.

$$\begin{matrix} G=m\cdot g \\ f=G\cdot\sin\theta=m\cdot g\cdot\sin\theta=\mu\cdot m\cdot g\cdot\cos\theta\\ \mu=\tan\theta\end{matrix}$$

The frictional coefficients of fine sandpaper, coarse sandpaper and ore particles were tested, which were 0.45, 0.7 and 0.95, respectively.

Based on the above method, the roughness of different materials was measured. In the physical experiment, the material with different friction coefficients is used to cover the surface of the footwall to achieve the change of the flatness of the footwall surface.

During the physical simulation experiment, the influence of the flatness of the footwall surface on the ore fluidity was evaluated by calculating the amount of ore remaining in the lower plate and the final recovery rate.

Thank you for your constructive and valuable review comments.

Sincerely yours,

Dr. Wu Jin
